# Benchmarking Perturbation-Based Saliency Maps for Explaining Atari Agents

**DOI:** 10.3389/frai.2022.903875

**Published:** 2022-07-13

**Authors:** Tobias Huber, Benedikt Limmer, Elisabeth André

**Affiliations:** Chair for Human-Centered Artificial Intelligence, University of Augsburg, Augsburg, Germany

**Keywords:** explainable reinforcement learning, explainable artificial intelligence (XAI), interpretable machine learning, deep reinforcement learning, feature attribution, saliency maps

## Abstract

One of the most prominent methods for explaining the behavior of Deep Reinforcement Learning (DRL) agents is the generation of saliency maps that show how much each pixel attributed to the agents' decision. However, there is no work that computationally evaluates and compares the fidelity of different perturbation-based saliency map approaches specifically for DRL agents. It is particularly challenging to computationally evaluate saliency maps for DRL agents since their decisions are part of an overarching policy, which includes long-term decision making. For instance, the output neurons of value-based DRL algorithms encode both the value of the current state as well as the expected future reward after doing each action in this state. This ambiguity should be considered when evaluating saliency maps for such agents. In this paper, we compare five popular perturbation-based approaches to create saliency maps for DRL agents trained on four different Atari 2,600 games. The approaches are compared using two computational metrics: dependence on the learned parameters of the underlying deep Q-network of the agents (sanity checks) and fidelity to the agents' reasoning (input degradation). During the sanity checks, we found that a popular noise-based saliency map approach for DRL agents shows little dependence on the parameters of the output layer. We demonstrate that this can be fixed by tweaking the algorithm such that it focuses on specific actions instead of the general entropy within the output values. For fidelity, we identify two main factors that influence which saliency map approach should be chosen in which situation. Particular to value-based DRL agents, we show that analyzing the agents' choice of action requires different saliency map approaches than analyzing the agents' state value estimation.

## 1. Introduction

With the rapid development of machine learning methods, Deep Reinforcement Learning (DRL) agents are making their way into increasingly high-risk applications, such as healthcare and robotics. However, this comes with an increasing complexity of state spaces and algorithms, making it hard if at all possible to comprehend the decisions of these agents (Heuillet et al., 2021). The research areas of Explainable Artificial Intelligence (XAI) and Interpretable Machine Learning aim to shed light on the decision-making process of such black-box models. In the case of DRL agents, which utilize neural networks with visual inputs, the most common explanation approach is the generation of saliency maps that highlight the most relevant input pixels for a given decision. In general, there are three main ideas on how to create saliency maps. The first idea is to use the gradient with respect to each input to see how much small changes of this input influence the prediction (Simonyan et al., 2014; Sundararajan et al., 2017; Selvaraju et al., 2020). The second group of methods uses modified propagation rules to calculate how relevant each neuron of the network was, based on the intermediate results of the prediction. Examples for this are Layer-wise Relevance Propagation (LRP) (Bach et al., 2015) or PatternAttribution (Kindermans et al., 2018). Finally, perturbation-based approaches perturb areas of the input and measure how much this changes the output of the network (Zeiler and Fergus, 2014; Ribeiro et al., 2016). Both gradient and modified propagation saliency maps have been applied to DRL agents (Zahavy et al., 2016; Huber et al., 2019). However, recent years saw a trend toward perturbation-based saliency maps (Greydanus et al., 2018; Puri et al., 2020). The major advantage of perturbation-based approaches is their model agnosticism. This means that they can be applied to any kind of reinforcement learning agent since they only use the in- and outputs of the agent.

If saliency maps are used to analyze DRL agents in high-risk applications, it is crucial that we can rely on the information provided by the saliency map. That is, the most relevant pixels according to the saliency map should actually be the most relevant pixels for the agent's strategy. This is often called fidelity of an explanation technique (Mohseni et al., 2021). The need for evaluating the fidelity of saliency maps was further demonstrated by Adebayo et al. (2018). They proposed sanity checks which showed that for some saliency map approaches, there is no strong dependence between the learned parameters of image classifiers and the saliency maps that analyze their underlying neural network. Surprisingly, there are no computational evaluations that assess and compare the fidelity of different saliency maps for DRL agents. This is despite the fact that DRL agents are more challenging to analyze than classification models (Heuillet et al., 2021). The decisions of a DRL agent are not isolated but are part of an overarching policy and might be influenced by delayed rewards, which may not be discernible in the current state. This makes it even more challenging to verify whether a saliency map matches the internal reasoning behind a DRL agent's action selection. In the prominent family of value-based DRL algorithms, for example, the output values do not only describe the the expected future reward after choosing each action. They also encode the estimated value of the input state for the current policy. This ambiguity is often ignored when saliency maps are applied to analyze the decisions of value-based DRL agents.

In this paper, we present, to the best of our knowledge, the first computational fidelity evaluation of different saliency maps for DRL agents. In particular, we make the following contributions. By focusing on five perturbation-based saliency map approaches, this work gives an overview of which approaches should be used in what situation by practitioners who do not have full access to their DRL agent's model. One drawback of perturbation-based saliency maps is that they depend on a choice of parameters for the saliency map approaches. To ensure that all of the algorithms tested in this paper perform reasonably well, we present a novel methodology to fine-tune the parameters of perturbation-based saliency maps for DRL agents. Furthermore, we propose a way to separately measure how well a saliency map captures an agent's respective action- and state-value estimation. We demonstrate that the performance of saliency map approaches differs considerably when measuring state-values compared to action-values.

As test-bed for our evaluation, we use the Atari 2600 environment. As metrics, we use the sanity checks proposed by Adebayo et al. (2018) and an insertion metric that measures if the most relevant pixels, according to the saliency map, actually affect the agent's decision. As far as we know, this is the first time that sanity checks are done for different perturbation-based saliency maps for any kind of model.

## 2. Related Work

In general, evaluation metrics for XAI approaches can be separated into two broad categories: human user studies and computational measurements (Mohseni et al., 2021). So far, DRL agents are mostly evaluated with user studies. Anderson et al. (2019) and Huber et al. (2021) conduct user studies to evaluate a single variant of modified propagation and perturbation-based saliency maps, respectively, with regards to mental models, trust, and user satisfaction. Puri et al. (2020) investigate whether perturbation-based saliency maps can help participants with chess puzzles, by highlighting which pieces were relevant for an agent's solution for these puzzles. Greydanus et al. (2018) test whether participants can identify overfit policies with the help of perturbation-based saliency maps. However, exclusively relying on user studies might only measure how convincing the saliency maps look but not how much they reflect the agent's internal reasoning. Therefore, it is important to additionally evaluate the fidelity of saliency maps through computational measurements (Mohseni et al., 2021). Such measurements also provide an easy way to collect preliminary data before recruiting users for a user study.

There is a growing body of work on computationally evaluating the fidelity of saliency maps for image classification models. The most common measurement is *input degradation*. Here, the input of the model is gradually perturbed, starting with the most relevant input features according to the saliency map. For visual input, this is either done by perturbing individual pixels per step (Ancona et al., 2018; Petsiuk et al., 2018) or by perturbing patches of the image in each step (Samek et al., 2017; Kindermans et al., 2018; Schulz et al., 2020). If the saliency map matches the model's reasoning, then the model's confidence should fall quickly. In addition to perturbing features, some newer approaches also propose an insertion metric where they start with fully perturbed inputs and gradually insert relevant features (Ancona et al., 2018; Petsiuk et al., 2018; Schulz et al., 2020). Recently, Tomsett et al. (2020) demonstrated that input degradation can be unreliable and is sensitive to implementation details like the type of perturbation. They conclude that researchers should employ several versions of this metric and try to understand potential reasons for unreliability.

A different technique to computationally evaluate saliency maps for classification models is to compare them with ground-truth saliency maps on modified datasets (Yang and Kim, 2019; Zhou et al., 2022). Here, a natural dataset is manipulated by adding artificial features that a model has to focus on to perfectly classify the dataset. Now, saliency maps for a perfect model on the manipulated dataset can be evaluated based on how well they localize the artificial features. However, it is not obvious how this method could be applied to DRL agents. First, in a reinforcement learning setting, there is no easily available dataset that can be manipulated. Secondly, DRL agents do not directly classify which objects or features are contained in an image. Therefore, it is not clear how the long-term decision-making of DRL agents is supposed to react to artificial features.

Another prominent computational measurement for saliency maps for image classification models are the so-called *sanity checks* proposed by Adebayo et al. (2018). These tests measure whether the saliency maps are dependent on the learned parameters of the model's neural network. One method for this is gradually randomizing the layers of the neural network and measuring how much this changes the saliency maps. If the saliency maps are faithful to what the network learned then they should change considerably for each randomized layer. Adebayo et al. did this for various gradient-based approaches and Sixt et al. (2020) additionally tested modified propagation methods. Both groups found that some approaches did not really depend on the parameters of the network and therefore cannot faithfully reflect the model's internal reasoning. As far as we know, there is no work that verified whether different types of perturbation-based saliency maps depend on the network's learned parameters for any kind of model even though this is one of the most popular saliency map approaches.

For DRL agents, there is very little work on computationally evaluating the fidelity of saliency maps. Puri et al. (2020) recorded which chess pieces human experts identified as important in a set of chess puzzles. This allows them to computationally compare these pieces to the pieces that saliency maps identify as relevant for an agent. However, this does not measure the saliency maps' fidelity to the agent's reasoning, but whether the saliency maps coincide with human reasoning. Huber et al. (2021) calculate sanity checks for a single modified propagation saliency map approach. Atrey et al. (2020) conduct experiments to verify hypotheses that are generated from observing saliency maps. However, both the formulation of hypotheses as well as their verification rely on manual inspection of the saliency maps. Therefore, this method requires extensive human effort. Moreover, it is not certain whether an erroneous hypothesis has been formulated because the saliency maps are faulty and do not reflect the agent's reasoning, or because the human observers misinterpreted the saliency maps. In this sense, we see our paper as the first computational evaluation to benchmark the fidelity of different saliency map approaches for DRL agents.

## 3. Methods

### 3.1. Test-Bed

The test-bed in our paper is the Atari Learning Environment (Bellemare et al., 2013). Four DRL agents were trained on the games MsPacman (simplified to Pac-Man in this work), Space Invaders, Frostbite, and Breakout using the Deep Q-Network (DQN) (Mnih et al., 2015) implementation of the OpenAI Baselines Framework (Dhariwal et al., 2017). We chose the DQN because it is the most basic DRL architecture which many other DRL agents build upon. The games were selected because the DQN performs very well on Breakout and Space Invaders but performs badly on Frostbite and Pac-Man. The agent observes the last 4 frames of the game and then chooses an action *a* from a pool of possible actions A. Hereby, each frame is down-sampled and greyscaled resulting in 84 × 84 × 4 input images. The reward is given by the change in in-game score since the last state, which we scaled such that the minimal possible reward is 1. All experiments were done on the same machine with an Nvidia GeForce GTX TITAN X GPU to ensure comparability of the results. Our code is available online^1^.

### 3.2. Saliency Map Approaches

As saliency map approaches, we chose Occlusion Sensitivity (Zeiler and Fergus, 2014) since it is the first and most basic perturbation-based saliency map approach. Furthermore, we use LIME (Ribeiro et al., 2016) and RISE (Petsiuk et al., 2018) which are two of the most popular perturbation-based saliency maps in general. Finally, we chose two approaches that were specifically proposed for DRL: Noise Sensitivity (Greydanus et al., 2018) and SARFA (Puri et al., 2020).

**Figure 1:**
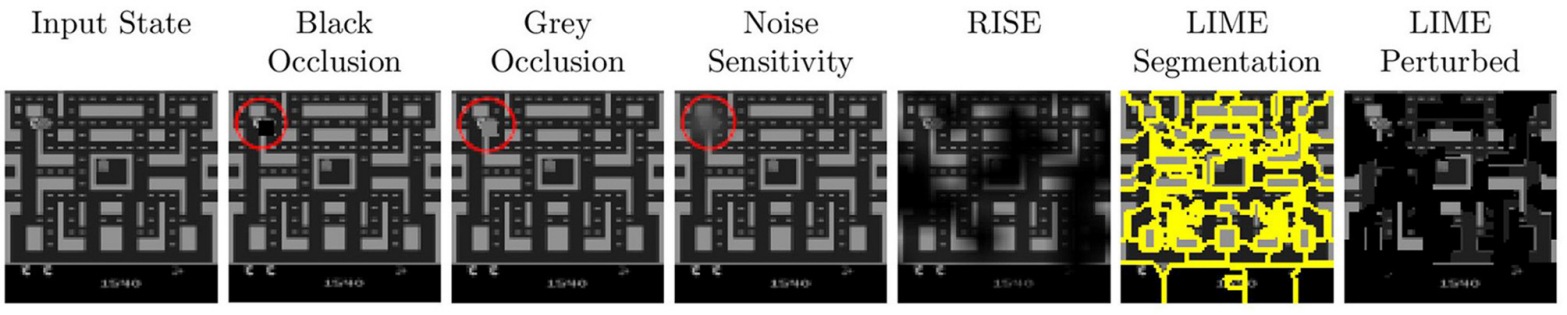
An example of the different types of perturbation used by the saliency map approaches in our work. The parameters are chosen in such a way that the idea of the perturbation can be easily identified. For Occlusion and Noise, the disturbed area is marked with a red circle.

The basic saliency map generation process is the same between all five approaches compared in this work. Let π be the agent that takes a visual input state *I* and maps it to a q-value *q*(*I, a*) for each possible action. To ease notation we use *q*(*I*) to describe the q-value of the action that should be analyzed. Most often this is the action with the highest q-value for the unperturbed input *I*, since this is the action that a fully trained agent would choose for *I*. An input image *I* with height *H* and width *W* can be defined as a mapping $I:\Lambda_{I}\to {\mathbb{R}}^{c}$ of each pixel λ∈Λ_*I*_ = {1, …, *H*} × {1, …, *W*} to *c* channels (e.g., *c* = 4 for the Atari environment which uses the channels to store the last 4 frames). To determine the relevance of each pixel λ for the prediction of the agent, all four approaches feed perturbed versions of *I* to the agent and then compare the resulting output values with the original results. However, the approaches widely differ in the way the image is perturbed (Figure 1) and how the relevance per pixel is computed:

**Occlusion Sensitivity Zeiler and Fergus (****2014****):** This approach creates perturbed states *I*′ by shifting a *n*×*n* patch across the original state *I* and occluding this patch by setting all the pixels within to a certain color (e.g., black). The relevance *S*(λ) of each pixel λ inside the patch is then computed based on the agent's confidence after the perturbation


(1) $$\begin{array}{r} S\left(\lambda \right)=1-q\left(I^{\prime }\right). \end{array}$$

Since the original source does not go into details about the algorithm, we use the *tf-explain* implementation as reference^2^. As long as the saliency maps are normalized this is equivalent to *q*(*I*)−*q*(*I*′) since all values in the saliency map are shifted by the same constant *q*(*I*)−1.

**Noise Sensitivity (Greydanus et al.**, **2018****):** Instead of completely occluding patches of the state, this approach adds noise to the state *I* by applying a Gaussian blur to a circle with radius *r* around a pixel λ. The modified state *I*′(λ) is then used to compute the relevance of the covered circle by comparing the agent's logit units π(*I*). For our DQN agents, π(*I*) is the vector of all q-values *q*(*I, a*) for each possible action:


(2) $$\begin{array}{r} S\left(\lambda \right)=\frac{1}{2}||\pi \left(I\right)-\pi \left(I^{\prime }\left(\lambda \right)\right)|{|}^{2} \end{array}$$

This is done for every *r*th pixel, resulting in a temporary saliency map smaller than the input. For the final saliency map, the result is up-sampled using bilinear interpolation.

**RISE (Petsiuk et al.**, **2018****):** This approach uses a set of *N* randomly generated masks {*M*_1_, …, *M*_*N*_} for perturbation. To this end, temporary *n*×*n* masks are created by setting each element to 1 with a probability *p* and 0 otherwise. These temporary masks are upsampled to the size of the input state using bilinear interpolation. The states are perturbed by element-wise multiplication with those masks *I*⊙*M*_*i*_. The relevance of each pixel λ is given by


(3) $$\begin{array}{r} S\left(\lambda \right)=\frac{1}{p\cdot N}\overset{N}{\underset{i=1}{\sum }}q\left(I⊙M_{i}\right)\cdot M_{i}\left(\lambda \right), \end{array}$$

where *M*_*i*_(λ) denotes the value of the pixel λ in the *i*th mask.

**LIME (Ribeiro et al.**, **2016****):** LIME uses image segmentation algorithms, like *SLIC, Quickshift* and *Felzenszwalb*, to divide the input state into superpixels (i.e., groups of pixels that share similar visual properties such as color). Then a dataset of *N* perturbed samples in the neighborhood of the input state is created. For each of those samples, a different combination of superpixels is “deleted” by setting all pixels within the superpixels to a certain value (we used 0). Using this dataset, an interpretable surrogate model is trained to predict the agent's decision based on the presence of superpixels. A common method for this surrogate model is linear regression. During training, the samples are weighted based on their proximity to the original input state. Finally, analyzing the weights of the trained surrogate model provides a relevance value for each superpixel.

**SARFA (Puri et al.**, **2020****):** This approach does not use a specific perturbation method. Puri et al. test noise perturbation for Atari games and occlusion for other domains. Given a perturbed state *I*′, SARFA measures the information specific to the action *a*′, which should be analyzed, by utilizing a softmax normalization $P\left(I,a^{\prime }\right):=\frac{exp\left(q\left(I,a^{\prime }\right)\right)}{\underset{a\in A}{\sum }exp\left(q\left(I,a\right)\right)}$ and calculating


(4) $$\begin{array}{r} \Delta p=P\left(I,a^{\prime }\right)-P\left(I^{\prime },a^{\prime }\right). \end{array}$$

To only measure relevant information, they additionally calculate


(5) $$\begin{array}{r} K=\frac{1}{1+KL\left(P_{rem}\left(I,a^{\prime }\right),P_{rem}\left(I^{\prime },a^{\prime }\right)\right)}, \end{array}$$

where *KL* is the Kullback-Leibler divergence and the vector $P_{rem}\left(I,a^{\prime }\right):=\frac{exp\left(q\left(I,a^{\prime }\right)\right)}{\underset{a\neq a^{\prime }}{\sum }exp\left(q\left(I,a\right)\right)}\forall a\neq a^{\prime }$ is the softmax over all outputs *except* the chosen action *a*′. The final relevance for each pixel that is perturbed in the state *I*′ is then given by:


(6) $$\begin{array}{r} S\left(\lambda \right)=\frac{2K\Delta p}{K+\Delta p} \end{array}$$

### 3.3. Metrics

We evaluate the generated saliency maps using two different computational metrics: Sanity checks and an insertion metric.

#### 3.3.1. Sanity Checks

The sanity checks proposed by Adebayo et al. (2018) measure the dependence between the saliency maps and the parameters learned by the neural network of the agent. To this end, the parameters of each layer in the network are randomized in a cascading manner, starting with the output layer. Every time a new layer is randomized, a saliency map for this version of the agent is created. The resulting saliency maps are then compared to the saliency map for the original network, using three different similarity metrics [Spearman rank correlation, Structural Similarity (SSIM), and Pearson correlation of the Histogram of Oriented Gradients (HOGs)]. If the saliency maps depend on the learned parameters of the agent then the saliency maps for the randomized models should vastly differ from the ones of the original model. Following Sixt et al. (2020), we account for saliency maps that differ only in sign by additionally computing the similarity metrics between the original saliency map and a version of each saliency map for the randomized models that was multiplied by −1. For each randomized model, we use the maximum of the similarity values with and without the −1 multiplication. For our tests, we calculate the sanity checks for 1, 000 states of each game.

Analogous to Adebayo et al. (2018), we calibrate the similarity metrics (Spearman rank correlation, SSIM, and Pearson correlation of the HOGs) such that high similarity values actually indicate similar saliency maps. Following Adebayo et al. (2018), we do this by calculating the similarity of 100 pairs of randomly generated saliency maps (Uniform and Gaussian). Since randomly sampled saliency maps should be very different on average, the mean of these similarities should be low. Using an SSIM window size of 7 and a HOG function with (3, 3) pixels per cell, two randomly sampled saliency maps with uniform distribution had mean similarity values (0.0087, 0.0136, 0.0096) and two random saliency maps with Gaussian distribution had mean similarity (0.0093, 0.0374, 0.0087).

#### 3.3.2. Insertion Metric

**Figure 2:**
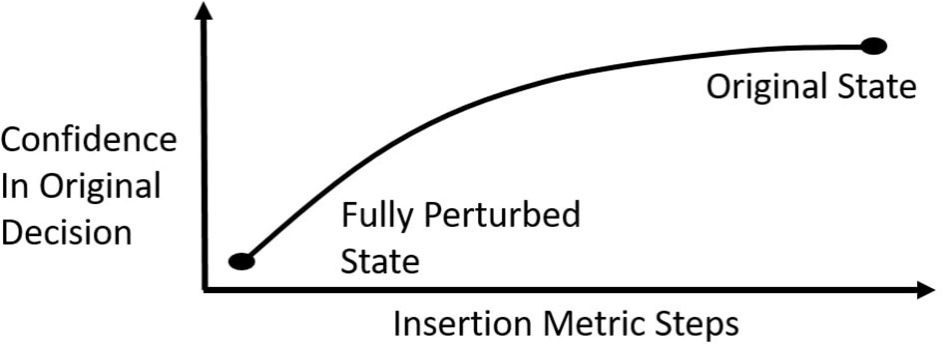
A schematic representation of the insertion metric curve.

If a saliency map is faithful to the agent, then the most relevant pixels should have the highest impact on the agent's decision. To test this property, we use an insertion metric similar to Petsiuk et al. (2018). We do not use a deletion metric, since we feel that it is too similar to the way that perturbation-based saliency maps are created. The insertion metric starts with a fully perturbed state. How this perturbation is done will be discussed in Section 3.3.2.1. In each step, 84 perturbed pixels (approximately 1.2% of the full state) are uncovered, starting with the most relevant pixels according to the saliency map. For LIME, the superpixels are sorted by their relevance but the order of pixels within superpixels is randomized. The partly uncovered state is then fed to the agent and its output for the action that the saliency map analyzes is measured. If the saliency map correctly highlights the most important pixels for this action, then the agent's output corresponding to this action should increase quickly for each partly uncovered image. Plotting the agent's output in each step of the insertion metric results in an insertion metric curve (Figure 2). If the output increases quickly, then the area under the insertion curve is high. Therefore, the Area Under the insertion metric Curve (AUC) is used to represent the result of the insertion metric for a single state.

Before we can apply the insertion metric to our DRL agents, we have to decide how to perturb the input and which output value we measure in each step.

##### 3.3.2.1. How to Perturb the Input

Tomsett et al. (2020) found that the choice of perturbation during the insertion metric has a high impact on the result of the metric. To be more robust against this influence, we use two different perturbations: black occlusion and uniform random perturbation in the range [0, 1]. Black is similar to the background color in most Atari games and therefore acts as “deleting” features from the state. Uniform random perturbation performed well for Tomsett et al. (2020).

##### 3.3.2.2. Which Output to Measure

Next, we have to decide which output we want to measure during the insertion metric. This comes with two further challenges.

First, the output q-values of value-based reinforcement learning algorithms like the DQN do not directly describe the agent's confidence in particular actions. Instead, they approximate the value of the current state in combination with each action. To disentangle this ambiguity, we propose to use two different sub-metrics. One measures how well the saliency map identifies features relevant to the state-value, and the other measures the same for the action value. For the state-value, we suggest using the q-value *q*(*I, a*) of the action that the saliency map is analyzing. For the action-value, we propose an estimation of the advantage as used by Wang et al. (2016):


(7) $$A(I,a)=q(I,a)-\frac{\sum_{a\in A}q(I,a)}{\left|A\right|}$$

The second challenge is that a reliable metric should not be distorted by outliers. For our Pac-Man agent, for example, we observed states with q-values around 1 and other states with q-values around 50. To reduce the effect of outlier states, we tested different methods of normalizing the agent's output during the insertion metric. The first normalization method we tested was inspired by Sixt et al. (2020) and forces each insertion curve to start at 0 and finish at 1. This is achieved by applying $f\left(x\right)=\frac{x-b}{t-b}$ to each insertion step result, where *b* is the output of the fully perturbed state and *t* is the output of the original state. As the second method, we only divided each insertion step by the output of the original state *t*. In this way, all insertion curves finish at the value of 1.

To identify which normalization method works best, we used 28 different variants of Occlusion Sensitivity saliency maps. The variants were obtained by varying the occlusion patch size between 4 and 10, using gray or black occlusion, and using the raw q-values or adding a softmax layer for the relevance calculation (Equation 1)^3^. For each variant, we calculated differently normalized insertion metrics over 1, 000 states of the Pac-Man environment for each of our two insertion metric perturbation methods. Tomsett et al. (2020) suggest using a low Standard Deviation (SD) as an early indicator for reliable saliency map metrics. Therefore, we chose the normalization method that resulted in the lowest SD of the area under the insertion curve across the 1, 000 states and both perturbation methods^4^. For each normalization method, Table 1 shows the highest and lowest SD among the 28 different Occlusion Sensitivity variants.

**Table 1:** The minimum and maximum SD when evaluating 28 different parameter combinations of Occlusion Sensitivity saliency maps with an insertion metric using different normalization functions.

**Normalization function**	**Minimum SD**	**Maximum SD**
**Measuring** ***Q*****-Values**		
No normalization	5.16	10.17
$f\left(x\right)=\frac{x}{t}$	1.14	2.06
$f\left(x\right)=\frac{x-b}{t-b}$	10.33	48.56
**Measuring advantage**		
No normalization	0.84	1.42
$f\left(x\right)=\frac{x}{t}$	1.99	3.78
$f\left(x\right)=\frac{x-b}{t-b}$	9.45	165.20

Interestingly, the full normalization to curves between 0 and 1 resulted in the highest SD. We think that this comes from the fact that our agents sometimes assign higher values to the fully perturbed state than to the original state. In these cases, *t*−*b* is negative, and applying *f*(*x*) inverts the insertion curve.

For the advantage, we obtained the lowest SD if we did not use any normalization. The q-values got the lowest SD when we divided each insertion metric step by the result of the original state.

##### 3.3.2.3. Final Setting

For our final evaluation of the different saliency map methods, we use 1, 000 states of each of the four Atari games. For each of those states, we calculated the insertion metric in four different variants: measuring the advantage of the chosen action with random and black perturbation, and measuring the normalized q-value with random and black perturbation.

### 3.4. Parameter Tuning

One of the biggest drawbacks of perturbation-based saliency map approaches is that they depend on a choice of parameters as can be seen in Section 3.2. Before we can run our final experiments we have to find suitable parameters. Section 3.4.3 will list the specific parameters that we tuned for each saliency map approach. This tuning is often done by manually adjusting the parameters until the resulting saliency maps look reasonable. However, tuning the parameters in this way does not guarantee that the saliency maps match the agent's internal reasoning. To obtain a fidelity benchmark for saliency maps, we computationally tune the parameters to perform well in the insertion metric. We do not tune the parameters for the sanity checks, since sanity checks do not measure how well a saliency map approach performs. Instead, they identify which approaches do not work at all. To tune the parameters for our final tests we need to decide on two things: how we combine the results from the four different insertion metric variants and which states we test the parameters on.

#### 3.4.1. Combining Insertion Metric Results

To combine the results of the random and black insertion metric variants, we measure the mean of the area under the insertion curve over both the black and the random perturbation insertion metric. For our evaluation, we would also like to find parameters that are able to analyze both the agent's action-value and state-value estimation. To this end, we standardize the mean AUC results of the aforementioned tests for the advantage and *q*-values measurements, respectively^5^. The sum of these standardized values is then used as a single value that measures the performance of the parameters. Parameters such as patch size have a strong influence on the run-time of the saliency map approaches. Therefore, to ensure comparability between approaches and to run our final experiment in a reasonable time, we did not select the top parameters. Instead, we use the best parameters that took up to three seconds to compute a single saliency map.

#### 3.4.2. Choosing a Test Set

As a test set for our parameter tuning, it is not feasible to use the full stream of 1, 000 states that we want to use in our final evaluation of the different saliency maps. LIME and RISE in particular have long computation times and a large number of possible parameter combinations (We will provide more information on the run-time of each saliency map approach in Section 4.3). This would make the run-time of the parameter test explode. Therefore, we need to find a suitable subset of states that represent as many states as possible. Since there are no test- or validation-sets in reinforcement learning we have to choose these subsets from the full stream of gameplay.

As potential candidates, we tested 22 different subsets consisting of 10 states each. Ten of these subsets were randomly selected and the other 12 subsets were selected by different variants of the HIGHLIGHT-DIV algorithm. The HIGHLIGHT-DIV algorithm selects a diverse set of states that give a good overview of the agent's policy (Amir and Amir, 2018). Hereby, it utilizes a diversity threshold that makes sure that the selected states are not too similar to each other. For this diversity threshold, we tested the 10, 20, 25, 28, 30, 32, 33, 35, and 40 percentile of the similarity values of the full 1,000 states stream^6^. To get even more diverse sets of states, we additionally tested two novel variations of HIGHLIGHTS-DIV: one variant where we used 5 of the most important and 5 of the least important states for the agents' strategy and one variant where we sorted all 1,000 states by importance and chose every 100th state to obtain states of all importance levels.

To compare how well these subsets represent the full stream of gameplay, we calculated the combined insertion metric results, as described above, for the full 1, 000 states of Pac-Man using 28 different parameter combinations of Occlusion Sensitivity. The particular parameters were chosen since they are fast to compute. Based on these results we obtained a “ground truth” for how those 28 parameters for Occlusion Sensitivity should be ranked. Now, a subset of states is suited for searching parameters if the parameter ranking obtained by the subset is similar to the ranking obtained by the full 1, 000 states. To calculate the similarity of different rankings we used both Spearman's and Kendall rank correlation coefficients. While this does not give conclusive evidence it gives a good estimation of which states do and do not work. The highest correlation to the ranking obtained by the full stream was achieved by the 30 percentile HIGHLIGHTS-DIV variant. For the action-value, the Spearman's rank correlation was 0.96 and the Kendall rank correlation was 0.85. For the state-value, the Spearman's rank correlation was 0.95 and the Kendall rank correlation was 0.81. The correlations for the other subsets can be seen in our repository^7^. It is important to note, that HIGHLIGHTS-DIV only performed well when the diversity threshold was very high. When the threshold was low the HIGHLIGHTS-DIV states performed worse than the random ones. We got the best results when the threshold was so high that increasing the threshold resulted in subsets with less than 10 states since the algorithm could not find any more states that could be added to the subset.

#### 3.4.3. Used Saliency Map Parameters

Using the combined insertion metric results and the test set described above, we tested a total of 4, 918 parameter combinations across all five saliency map methods. The full results of our tests for each method can be viewed in our repository^8^.

##### 3.4.3.1. Occlusion Sensitivity

For Occlusion Sensitivity, we tested patches of size 1 to 10, black and gray occlusion color, and whether applying a softmax layer to the output q-values before creating the saliency map improves results. The top 10 results are shown in Table 2.

**Table 2:** Best parameters for Occlusion Sensitivity.

**AUC**	**Patch size**	**Color**	**Softmax**	**Time**
6.76	1	Black	No	10.94
3.44	1	Gray	No	11.09
3.42	1	Black	Yes	11.51
3.03	1	Gray	Yes	11.50
**2.33**	**2**	**Black**	**No**	**2.80**
0.93	2	Black	Yes	2.88
0.32	3	Black	No	1.26
0.26	2	Gray	No	2.83
0,04	2	Gray	Yes	2.87
−0.06	4	Black	No	0.69

*The final parameters are marked in bold*.

##### 3.4.3.2. Noise Sensitivity and SARFA

For Noise Sensitivity, we tested circles with a radius of 1 to 10. The top ten parameters are shown in Table 3A. SARFA was not introduced with a specific perturbation method. Analogous to Puri et al., we test blurred circles of radius 1–10 as used in Noise Sensitivity. Additionally, we also use circles that are occluded with black color. The top ten results are shown in Table 3B.

**Table 3:** Best parameters for Noise Sensitivity **(A)** and SARFA **(B)**.

**AUC**	**Radius**	**Time**
**(A)**		
3.08	2	5.79
2.21	1	22.84
**0.94**	**3**	**2.62**
0.68	9	0.38
0.48	10	0.31
−0.05	4	1.48
−0.49	8	0.44
−0.84	5	0.99
−2.07	6	0.68
−3.94	7	0.51
**AUC**	**Radius**	**Perturbation**	**Time**
**(B)**			
7.03	1	Black	12.05
**1.46**	**2**	**Black**	**3.00**
1.09	1	Blur	23.70
0.57	8	Blur	0.46
0.55	2	Blur	6.12
0.49	9	Blur	0.39
0.40	10	Blur	0.32
0.27	3	Black	1.40
0.08	3	Blur	2.77
0.01	5	Blur	1.06

*The final parameters are marked in bold*.

##### 3.4.3.3. RISE

For RISE we tested 500, 1, 000,…,3, 000 masks of size 4 to 24. The probability *p* with which each pixel is occluded varied between 0.1 and 0.9 in steps of 0.1. Analogous to Occlusion Sensitivity, we also investigated whether it makes sense to add a softmax layer after the output during the saliency map creation. The top five results are shown in Table 4.

**Table 4:** Best parameters for RISE.

**AUC**	***p***	**Mask size**	**Masks**	**Softmax**	**Time**
3.21	0.8	11	3,000	Yes	5.09
3.04	0.7	13	3,000	No	4.76
2.99	0.9	24	2,500	Yes	3.98
2.94	0.8	4	3,000	No	4.66
⋮	Skipping 9 parameters that took more then 3 s.				
**2.66**	**0.5**	**8**	**1,000**	**No**	**1.54**

*The final parameters are marked in bold*.

##### 3.4.3.4. LIME

For LIME we tested the three most common Segmentation techniques *SLIC, Quickshift* and *Felzenszwalb* and varied the number of samples on which the local interpretable model is trained. For the number of samples, we took the default number of samples (1, 000) and increased it in steps of 500 up to 3, 000. To determine which parameter ranges we should explore for each segmentation algorithm, we performed preliminary tests where we visually checked which parameters resulted in different segmentation. For Felzenszwalb segmentation we used a scale factor of 1,21,…,101, a minimum component size from 1 to 8 and Gaussian smoothing kernels with width σ of 0,0.25,…,1. The top results are shown in Table 5. For SLIC we tested 40,60 to 240 segments, a compactness factor of 0.001,0.01,…,10 and Gaussian smoothing kernels with width σ of 0,0.25,…,1. The top five parameter combinations can be seen in Table 6. Finally, we tested Quickshift with a color ratio of 0.0,0.33,0.66 and 0.99, a kernel size from 1 to 6 and a max distance of kernelsize*i, where *i* goes from 1 to 4. The top results are shown in Table 7.

**Table 5:** Best parameters for LIME with Felzenszwalb segmentation.

**AUC**	**Scale**	**Sigma**	**Minimum size**	**Num samples**	**Time**
4.35	21	0.5	0	3,000	10.73
3.58	21	0.75	2	3,000	7.38
3.53	1	1.0	0	2,000	22,03
3.29	21	0.5	0	2,500	8.95
⋮	Skipping 14 parameters that took more then 3 s.				
**2.55**	**21**	**0.5**	**4**	**1,000**	**1.71**

*The final parameters are marked in bold*.

**Table 6:** Best parameters for LIME with SLIC segmentation.

**AUC**	**Num segments**	**Compactness**	**Sigma**	**Num samples**	**Time**
3.99	200	10.0	1.0	3,000	3.13
**3.86**	**200**	**10.0**	**0.25**	**2,000**	**2.08**
3.48	200	10.0	0.0	3,000	3.11
3.46	200	0.001	0.25	3,000	2.36
3.44	200	10.0	0.5	1,000	1.06

*The final parameters are marked in bold*.

**Table 7:** Best parameters for LIME with Quickshift segmentation.

**AUC**	**Kernel size**	**Max distance**	**Ratio**	**Num samples**	**Time**
6.24	1	1	0.0	3,000	11.38
4.97	1	1	0.0	2,500	9.57
4.80	1	2	0.0	2,500	4.46
4.50	1	2	0.0	3,000	5.39
**4.23**	**1**	**2**	**0.0**	**1,500**	**2.75**

*The final parameters are marked in bold*.

## 4. Results

**Figure 3:**
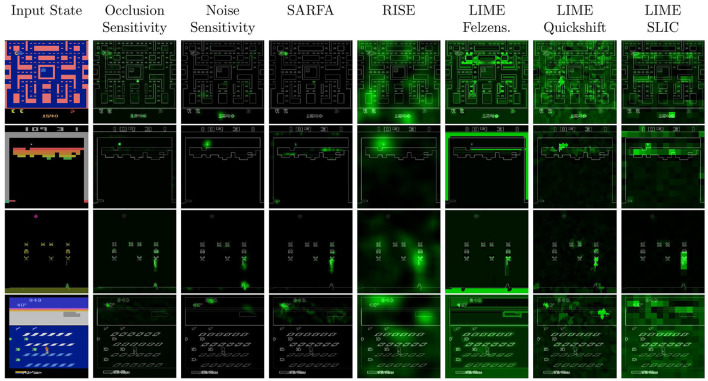
Example saliency maps for games we tested. From top to bottom: Pac-Man, Breakout, Space Invaders, and Frostbite. For a better visibility, the saliency maps are displayed in green color over a simplified version of the states. The higher the intensity of the green color, the higher the relevance of the corresponding pixel for the agent's decision.

Figure 3 shows example saliency maps for all four games used in our experiments. To prevent cherry-picking of particularly convincing states, the states are chosen by the HIGHLIGHTS-DIV algorithm (Amir and Amir, 2018).

### 4.1. Sanity Checks

**Figure 4:**
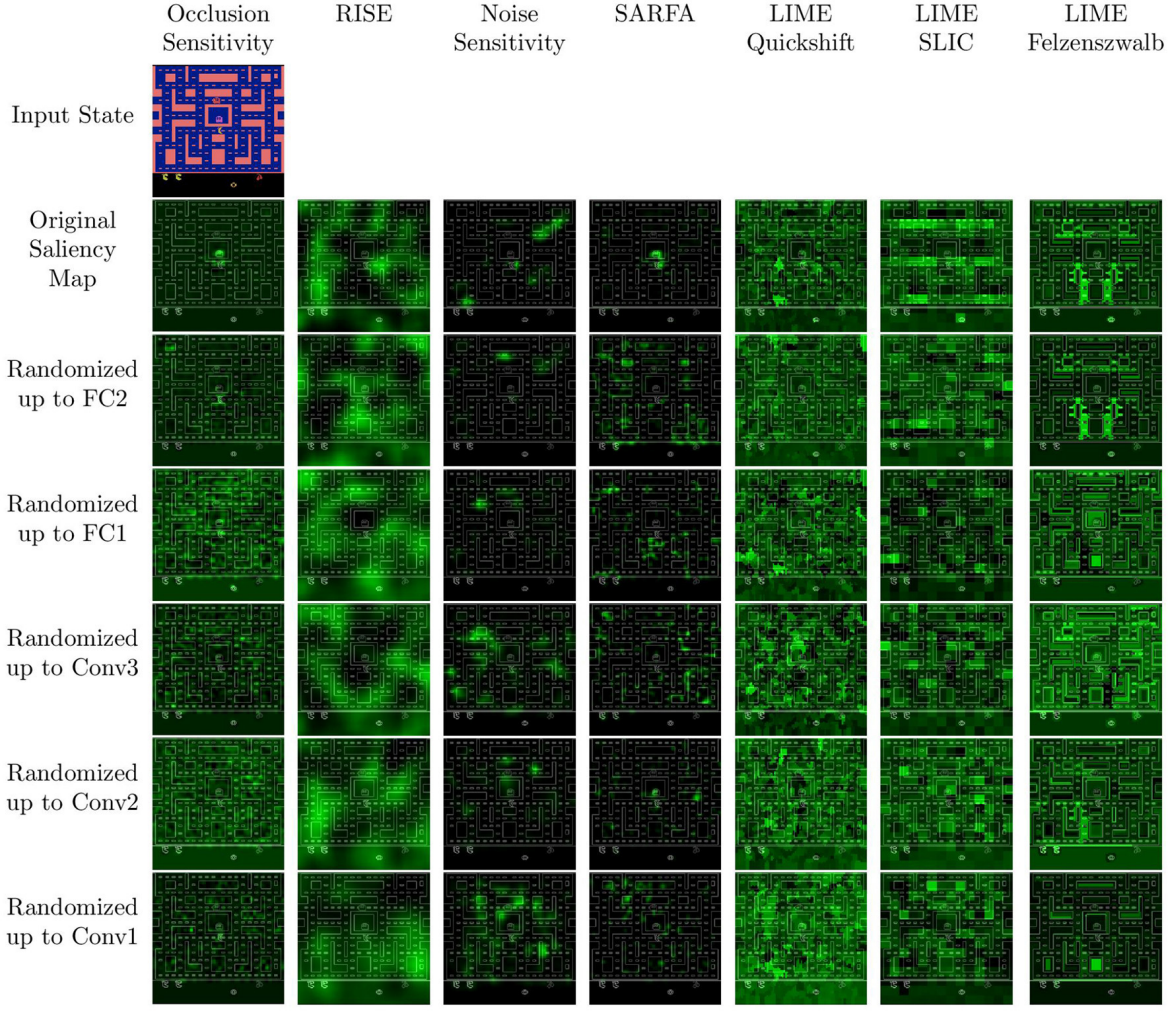
Example saliency maps for the parameter randomization sanity check. From top to bottom each row after the first is generated for agents with cascadingly randomized layers starting with the output layer.

**Figure 5:**
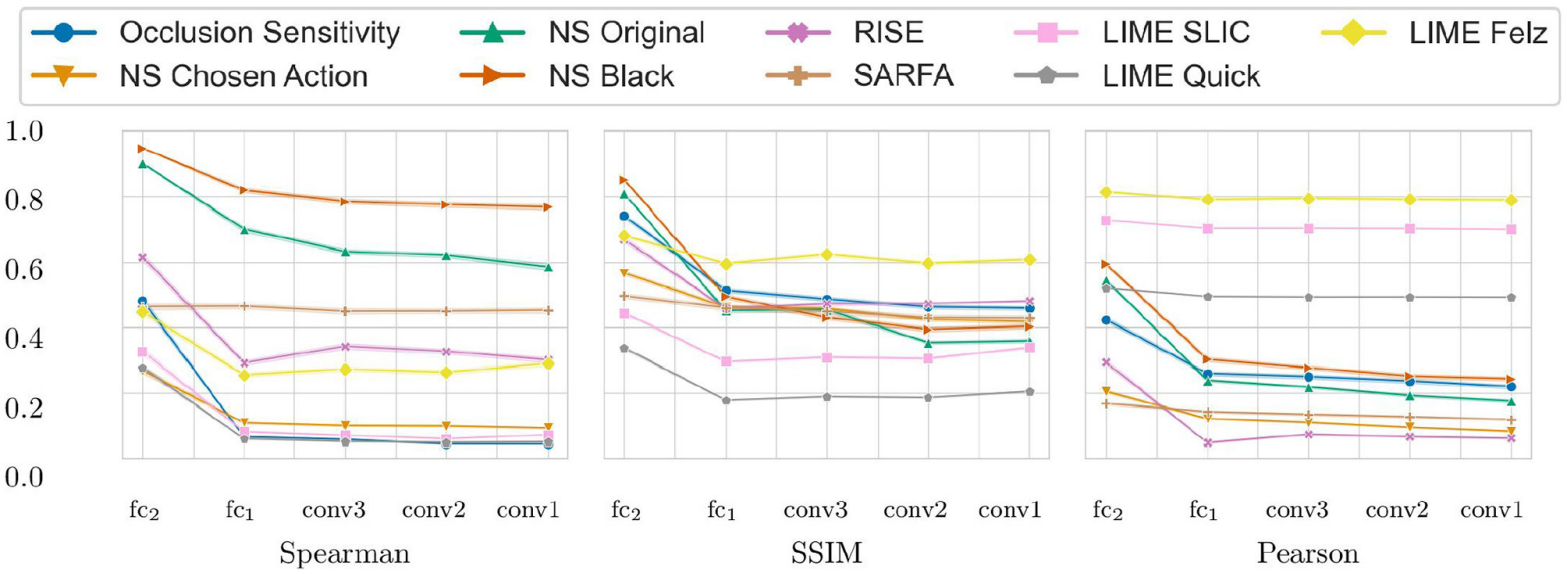
Results of the sanity checks for the different saliency map approaches (*NS* is noise Sensitivity). Measured for 1, 000 states of each of the 4 tested games. Starting from the left, each mark represents an additional randomized layer starting with the output layer. The y-axis shows the average similarity values (Spearman rank correlation, SSIM, Pearson correlation of the HOGs). High values indicate a low parameter dependence. The translucent error bands show the 99% CI but are barely visible due to low variance in the results.

**Figure 6:**
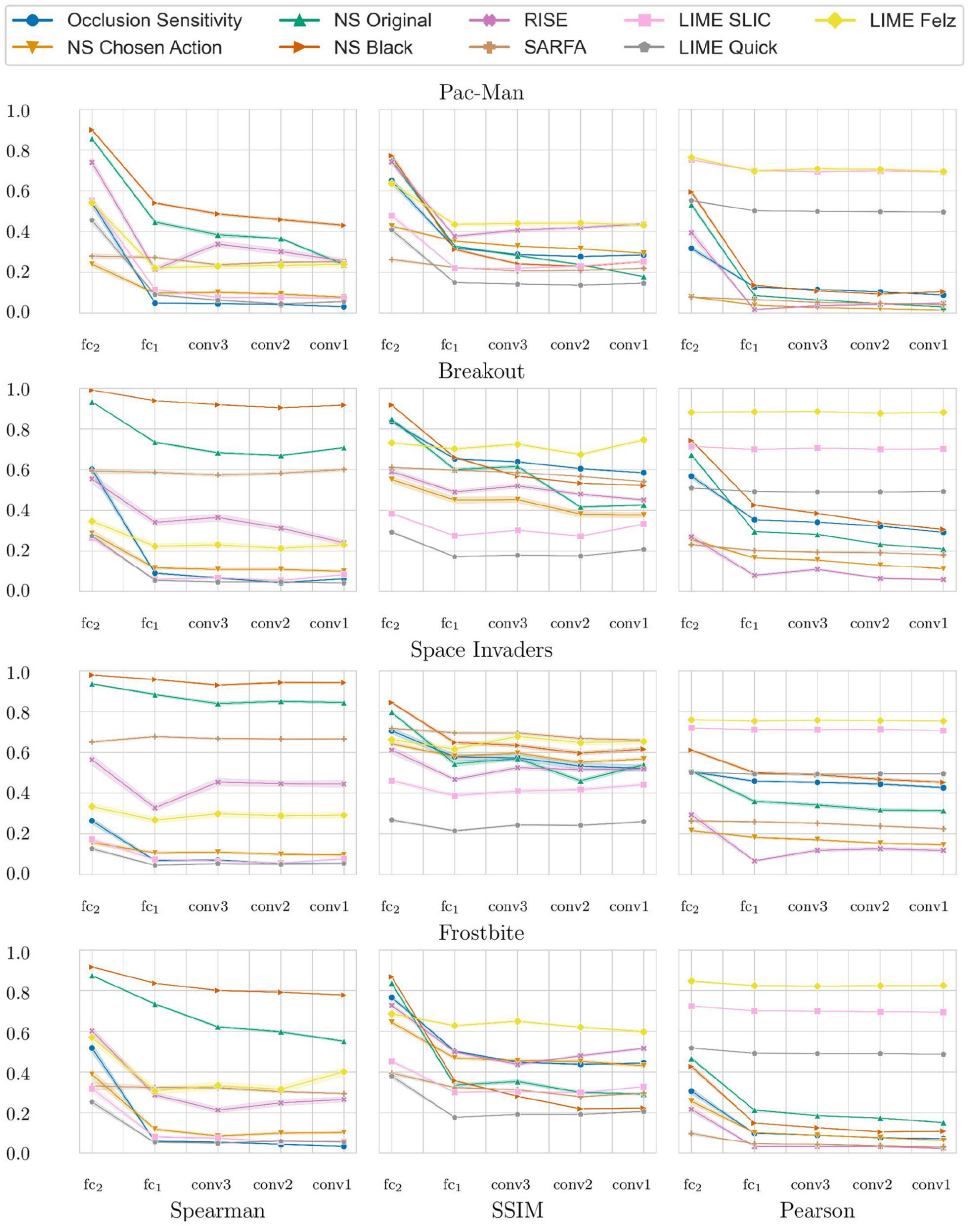
Results of the sanity checks for each individual game for the different saliency map approaches (*NS* is noise Sensitivity). Measured for 1, 000 states of each of the 4 tested games. Starting from the left, each mark represents an additional randomized layer starting with the output layer. The y-axis shows the average similarity values (Spearman rank correlation, SSIM, Pearson correlation of the HOGs). High values indicate a low parameter dependence. The translucent error bands show the 99% CI.

An example of the different saliency maps during a single run of the sanity check can be seen in Figure 4. The combined results of the sanity checks test are shown in Figure 5. The results for each individual game can be seen in Figure 6. The lower the scores the higher the dependence on the agents' learned parameters. Notably, LIME has a very high Pearson correlation of HOGs. Furthermore, the original Noise Sensitivity has low dependence on the parameters of the output layer when compared to Occlusion Sensitivity. Since those two approaches are very similar in theory, we implemented two modifications of Noise Sensitivity to investigate the reason for this difference in parameter dependence. First, *Noise Sensitivity Black* occludes the circles in the Noise Sensitivity approach with black color instead of blurring them. Second, *Noise Sensitivity Chosen Action* changes the way that the relevance of each pixel is calculated from the original equation (Equation 2), which takes all actions into account, to the one used by Occlusion Sensitivity (Equation 1), which focuses on the chosen action. We did not test a combination of black circles and the Occlusion Sensitivity relevance calculation since that would be equivalent to Occlusion Sensitivity with circles instead of squares. While the black occlusion did not really change the sanity check results, the change of the relevance calculation immensely increased the dependence on the learned parameters.

### 4.2. Insertion Metric

Table 8 reports the sample mean and SD of the insertion metric results for 1,000 states of each game and each saliency map approach^9^. To get a baseline performance, we also calculated the insertion metric with uniformly sampled random saliency maps. For some games and sub-metrics, the mean area under the insertion curve is negative. This is due to the fact that some agents assign high negative q-values and advantages to the fully perturbed state. For most games, RISE has the best results for measuring the raw q-values on random perturbation. However, the results for measuring advantage with random perturbation are poor for all approaches. For Frostbite and Space Invaders, and measuring the advantage with random perturbation, the random saliency maps even performed better than all other approaches. For the other two games, RISE has the highest values. When using black color perturbation during the insertion metric, Occlusion Sensitivity obtained very good results for measuring the state-value, and SARFA worked best for the advantage. However, their results for random perturbation were very poor. From our parameter tuning, we knew that this depended on the color of perturbation used during the saliency map generation. Therefore, we additionally tested Occlusion Sensitivity with gray color and SARFA with noise perturbation as used by Noise Sensitivity. The other parameters remained unchanged. Table 9 shows the results of those additional tests. Notably, Occlusion Sensitivity got the highest q-value random insertion results in Pac-Man, Frostbite, and Space Invaders. SARFA got the best advantage results for random insertion for Pac-Man and Frostbite only slightly losing to Occlusion Sensitivity with gray color in Space Invaders. The performance of both approaches on black perturbation fell to a level similar to the random baseline. The exception to most observations described above is Breakout. Here, the LIME variants performed the best across most metrics. SLIC segmentation in particular achieves at least the second-highest score in each metric. It is worth noting, that this game also has the highest SD values.

**Table 8:** The sample mean and SD of the insertion metric curve for 1, 000 states of each game.

**Metric**	**Occlusion**	**Noise**	**SARFA**	**RISE**	**LIME Felz**.	**LIME Quick**.	**LIME SLIC**	**Baseline**
**Pac-Man:**								
Q-val rand	0.54 ± 1.3	0.75 ± 0.7	0.76 ± 1.2	**1.1** **±2.0**	0.46 ± 0.7	0.67 ± 1.1	0.62 ± 1.1	0.85 ± 1.5
Adv rand	–0.52 ± 1.2	–0.03 ± 0.8	–0.74 ± 1.3	**–0.01** **±1.1**	–0.43 ± 1.2	–0.44 ± 1.0	–0.36 ± 1.1	–0.22 ± 1.0
Q-val black	**3.08** **±3.2**	0.66 ± 0.8	0.83 ± 1.8	1.01 ± 1.8	2.83 ± 5.3	2.49 ± 4.7	2.47 ± 4.4	0.53 ± 0.8
Adv black	1.23 ± 1.6	0.15 ± 0.3	**1.7** **±0.8**	0.21 ± 0.4	0.64 ± 0.7	0.94 ± 0.5	0.67 ± 0.5	0.06 ± 0.3
**Breakout:**								
Q-val rand	–0.72 ± 2.5	–1.01 ± 3.0	–3.19 ± 3.9	–0.97 ± 2.7	–0.98 ± 2.7	**–0.48** **±4.1**	–0.53 ± 3.2	–2.21 ± 2.9
Adv rand	–0.42 ± 4.7	–1.52 ± 8.4	–0.92 ± 8.4	**0.85** **±6.1**	–0.7 ± 6.5	–0.54 ± 5.4	–0.05 ± 4.8	–0.76 ± 5.8
Q-val black	3.16 ± 4.2	3.04 ± 4.2	1.97 ± 2.0	3.39 ± 4.2	**7.48** **±9.6**	5.8 ± 8.7	6.12 ± 9.7	2.13 ± 3.1
Adv black	0.02 ± 0.5	0.19 ± 0.6	0.53 ± 1.1	0.29 ± 0.6	0.24 ± 0.6	0.24 ± 0.4	**0.71** **±1.4**	0.07 ± 0.2
**Frostbite:**								
Q-val rand	0.56 ± 1.0	0.83 ± 1.0	0.73 ± 1.0	**0.92** **±1.1**	0.75 ± 0.9	0.37 ± 1.0	0.36 ± 1.0	0.88 ± 1.1
Adv rand	0.31 ± 1.1	0.38 ± 1.2	0.2 ± 1.2	0.35 ± 0.9	0.2 ± 0.9	0.24 ± 1.3	0.23 ± 1.3	**0.4** **±1.2**
Q-val black	**5.65** **±3.1**	0.58 ± 0.2	1.53 ± 1.6	2.4 ± 1.7	2.71 ± 2.4	5.12 ± 4.1	3.25 ± 2.5	0.51 ± 0.4
Adv black	0.59 ± 0.9	0.2 ± 0.2	**1.22** **±0.9**	0.25 ± 0.3	0.26 ± 0.3	0.28 ± 0.4	0.26 ± 0.3	0.16 ± 0.2
**Space Invaders:**								
Q-val rand	–0.7 ± 0.6	–0.6 ± 0.6	–0.8 ± 0.6	**–0.39** **±0.4**	–1.12 ± 0.9	–0.81 ± 0.7	–0.88 ± 0.7	–1.1 ± 0.8
Adv rand	0.76 ± 3.5	0.83 ± 3.4	0.79 ± 3.7	0.66 ± 2.8	0.87 ± 4.3	0.76 ± 3.7	0.87 ± 3.6	**0.89** **±4.2**
Q-val black	1.01 ± 0.2	0.73 ± 0.1	0.74 ± 0.2	0.89 ± 0.1	1.02 ± 0.2	1.08 ± 0.2	**1.11** **±0.3**	0.56 ± 0.1
Adv black	0.28 ± 0.4	0.26 ± 0.3	**0.59** **±0.4**	0.21 ± 0.2	0.24 ± 0.2	0.25 ± 0.2	0.29 ± 0.2	0.13 ± 0.2

*Q-val and Adv measure the change of the normalized q-value and advantage, respectively. Rand and black use random and black perturbation, respectively, during the insertion metric. The highest values are marked in bold*.

**Table 9:** The sample mean and SD of the insertion metric curve for our additional experiments with different perturbations for Occlusion Sensitivity and SARFA.

**Metric**	**Occlusion gray**	**SARFA blur**
**Pac-Man:**		
Q-val rand	**2.98** **±3.5**	1.0 ± 2.2
Adv rand	0.44 ± 1.8	**1.12** **±1.0**
Q-val black	0.32 ± 0.2	0.62 ± 1.3
Adv black	–0.13 ± 0.3	0.23 ± 0.4
**Breakout:**		
Q-val rand	–0.83 ± 2.6	–0.8 ± 3.4
Adv rand	0.4 ± 5.3	–0.4 ± 5.8
Q-val black	1.99 ± 2.5	3.0 ± 3.9
Adv black	0.11 ± 0.5	0.21 ± 0.7
**Frostbite:**		
Q-val rand	**3.54** **±2.3**	1.13 ± 1.2
Adv rand	0.66 ± 1.1	**0.73** **±1.2**
Q-val black	0.5 ± 0.5	0.58 ± 0.3
Adv black	0.16 ± 0.2	0.3 ± 0.3
**Space Invaders:**		
Q-val rand	**0.07** **±0.7**	–0.75 ± 0.7
Adv rand	**1.04** **±3.5**	1.02 ± 3.7
Q-val black	0.48 ± 0.2	0.66 ± 0.2
Adv black	0.12 ± 0.3	0.44 ± 0.4

*Q-val and Adv measure the change of the normalized q-value and advantage, respectively. Rand and black use random and black perturbation, respectively, during the insertion metric. The bold values beat the highest values for the respective metric in our original experiment*.

### 4.3. Run-Time Analysis

The run-time of an algorithm can be an important aspect when choosing between different approaches. We computed the mean time it took each algorithm to create a single saliency map using the *timeit* python library. To get a feeling of how this is affected by different parameters of the saliency map approaches, we measured the time during our parameter tuning process where each parameter combination was used on 10 different states (see Section 3.4 for the full results).

The fastest approach was Occlusion Sensitivity which uses simple color occlusions followed by the more complex blur perturbation of SARFA and Noise Sensitivity. However, this was strongly dependent on the size of the perturbation patches and circles, respectively. Using a patch size or radius of 1, these approaches were among the slowest with a mean run-time of around 22*s* for the blur perturbation and approximately 11*s* for the black occlusion variant. However, increasing the patch size and radius to 2 already drastically reduced the run-time. For RISE, the run-time mainly depends on the number of masks. With 3, 000 masks the run-time was always close to 5*s* per saliency map. However, compared to the aforementioned saliency map approaches, this did only decrease slowly when decreasing the number of masks. Thus, the average and the fastest run-time were much slower for RISE than for SARFA, and Occlusion and Noise Sensitivity. The slowest approach we tested was LIME. However, this was strongly influenced by the number of segments that the segmentation functions generated and the number of learning steps for the locally interpretable classifier. For SLIC, which creates relatively big segments, LIME was quite fast with a maximum run-time of 3.87*s* with the slowest parameters. In contrast, the run-time for Felzenswalb easily exploded and reached a maximum of 33.64*s* per saliency map. Quickshift was in the middle of those two approaches with a maximum run-time of 12.50*s* which did not decrease as quickly as the run-time of Occlusion and Noise Sensitivity, and SARFA.

## 5. Discussion

### 5.1. Sanity Checks

The results of our sanity checks show that most of the perturbation-based saliency map approaches tested in this paper are dependent on the learned parameters of the agent's neural network. Their dependence on the learned parameters is generally comparable to the best gradient-based approaches tested by Adebayo et al. (2018) and the best modified propagation approaches tested in Sixt et al. (2020). The only exceptions to this are Noise Sensitivity and LIME.

Noise Sensitivity showed little dependence on the parameters of the output layer (Figure 5). Since the output layer has the highest impact on the actual decision of a network, it is crucial that a faithful saliency map depends on the weights learned in this layer. Our results empirically show that replacing the original equation of Noise Sensitivity to calculate the relevance of each pixel with the equation used by Occlusion Sensitivity greatly increases the parameter dependence. We think that this is due to the fact that the original equation takes all actions into account and therefore measures a general increase in entropy within the activations of the output layer. In contrast, Occlusion Sensitivity only measures the action which is actually analyzed and therefore captures a more specific change in the output layer activation. Recently, Puri et al. (2020) also criticized that the saliency maps by Greydanus et al. (2018) take all actions into account. The results of our sanity checks provide the first computational evidence for this critique.

LIME performed well in the sanity check measurements using SSIM and Spearman correlation. Only the Pearson correlation of the HOGs was very high between LIME saliency maps for the trained and randomized agents. However, the reason for this is not necessarily a low dependence on the agent's learned weights. More likely it is due to the fact that all LIME saliency maps for a given state work with the same superpixels. Since every pixel inside a superpixel has the same value there are hard edges between the superpixels. These edges are captured by the HOGs and result in high values of the Pearson correlation of the HOGs.

### 5.2. Insertion Metric

During our parameter tuning, we tried our best to find parameters that result in saliency maps that work for both black and random perturbation and capture both the agent's action-value as well as state-value estimation. Despite these efforts, no saliency map approach performed well across all sub-metrics. The best results for measuring the state-value were obtained by Occlusion Sensitivity and the best results for the action-value were obtained by SARFA. This distinction is illustrated by the fact that no SARFA saliency map for Pac-Man, which we looked at, identified the in-game score as relevant (e.g., Figure 3). The score is a good indicator for the value of the current state and is frequently highlighted by all other approaches we tested. However, based on the rules of the game, it is not necessary to know the score to choose the correct action in a given Pac-Man state.

Additionally, the saliency maps' fidelity depended on the type of perturbation. The area under the insertion curve with black perturbation was the highest when the saliency map approaches used black occlusion. To mitigate this effect some saliency map approaches utilize blurring during their perturbation. Surprisingly, this was also sensitive to the perturbation type of the insertion metric in our tests. Similar to gray occlusion, blurring performed best for the random perturbation insertion metric and did not do well on black perturbation. The closest thing to a saliency map approach that fits all sub-metrics was RISE. However, the results here were considerably worse than the results for Occlusion Sensitivity and SARFA with parameters that fit the respective sub-metric, especially when analyzing the action-value estimation.

These results do not necessarily mean that the evaluated saliency map methods are not suited to explain DRL agents. However, they demonstrate that none of the approaches answers the general question: “What was the most relevant input region for the agent's decision?”. Instead, they answer more specific questions depending on the type of perturbation and whether the state or action value is analyzed. For example, SARFA with black perturbation for Pac-Man is suited to answer the question: “The presence of which objects was relevant for the agent's choice of action.” Since the black background color of Pac-Man acts as deleting objects and SARFA measures the action advantage. In contrast, Occlusion Sensitivity with black color would answer the same question with regard to the agent's evaluation of the current state. Based on this, we advise future researchers to clearly define what question they want to investigate. Depending on that question, a fitting saliency map method can be chosen.

Our parameter tuning experiments also showed that the fitting saliency map method can strongly depend on single parameters of the saliency map methods. Therefore, we encourage future researchers to conduct systematic parameter searches fitting their question similar to the one described in this paper. Manually adjusting the parameters until the resulting saliency maps look reasonable might lead to saliency maps that look convincing but do not match the agent's internal reasoning.

### 5.3. Limitations

We used four different variants of the insertion metric to get a good estimate of saliency map approaches' fidelity in different situations. Between those variants, we already found distinct differences. This fact reinforces the findings by Tomsett et al. (2020) that current fidelity metrics for saliency maps can be very sensitive to specifics of their implementation. For value-based RL in particular, we extend the results of Tomsett et al. by demonstrating that there are also considerable differences between metrics that measure the action-value and metrics that measure the state-value. However, it can not be ruled out that other fidelity metric variants might result in even more insights. To ease future evaluations and parameter searches, a great challenge for XAI research will be the development of more general fidelity metrics for saliency maps.

Another potential limitation of our results is that recent work indicates that simply displaying saliency maps to end-users might not be suited as a final explanation (Danesh et al., 2021; Huber et al., 2021). However, saliency maps are still often used as primary components of more sophisticated explanation frameworks (e.g., Danesh et al., 2021). We argue that it is even more crucial to evaluate the fidelity of saliency maps in situations where their information is used as an integral component of more complex explanation mechanisms.

We only used DRL agents with visual input in our evaluation since this is the most common application for saliency maps. It is possible to apply saliency map methods to DRL agents with other input domains as used in 3D locomotion tasks (Todorov et al., 2012), queueing network controls (Dai and Gluzman, 2022), and recommendation systems (Zhao et al., 2021). In this context, the saliency map methods are often referred to as Feature Attribution methods. This raises the question of whether our results extend to Feature Attribution methods in non-visual domains. Since visual image manipulations (e.g., image segmentation and Gaussian noise) do not make sense in non-visual input domains, Feature Attribution methods use different input perturbations in non-visual domains. Apart from that, the saliency map methods discussed in this work can be directly applied to any agent with discrete action space. Continuous action spaces require further adjustments. Therefore, our findings that are not related to the input perturbation should still apply to DRL agents with discrete action spaces in non-visual domains. This includes the difference between analyzing the agent's action-value and state-value estimation, as well as the parameter independence of the relevance calculation of Noise Sensitivity.

## 6. Conclusion

This paper compared five different perturbation-based saliency map approaches measuring their dependence on the agent's parameters and their fidelity to the agent's reasoning. Our main findings are:

- Most of the approaches tested in this work do depend on the agent's learned parameters. Only Noise Sensitivity showed less dependence on the learned parameters of the output layer. We empirically show that this is due to Noise Sensitivity's original relevance calculation. Replacing this calculation with a calculation that only takes the analyzed action into account, drastically increases the dependence on the parameters of the output layer.
- For value-based DRL agents, there are considerable differences between analyzing the agent's action-value and state-value estimation. While this distinction is hidden within the agent's output q-values, future practitioners should be aware of which of the two they want to analyze and choose their saliency maps accordingly. To investigate how well saliency maps for value-based DRL agents capture this distinction, we proposed an adjustment to existing input degradation metrics for image classifiers. In our tests, SARFA worked best to capture the action-value while Occlusion Sensitivity and RISE were more suited for the state-value.
- Depending on which perturbation method the approaches use, the resulting saliency maps only analyze how sensitive the agent is with regard to specific types of perturbation. While this seems obvious, it was true even for perturbation methods that utilized blurring specifically to reduce their dependence on a choice of occlusion color. In contrast to the action- and statue-value distinction, this is not an inherent property of the DRL agents but might be seen as a flaw of current perturbation-based saliency map approaches. Our results demonstrate that there is still a need to further develop perturbation-based saliency map approaches. For now, researchers have to decide which types of perturbation are meaningful and interesting for their application. Based on this, they can choose an appropriate perturbation method. For example, by performing a parameter search similar to the one conducted in this work.

## Data Availability Statement

The datasets presented in this study can be found in online repositories. The names of the repository/repositories and accession number(s) can be found under the following link: https://github.com/belimmer/PerturbationSaliencyEvaluation.

## Author Contributions

TH oversaw the development and wrote major parts of the paper and code. BL wrote major parts of the code and certain sections of the paper. EA supervised the entire work as well as the drafting of the article. All authors contributed to manuscript revision, read, and approved the submitted version.

## Conflict of Interest

The authors declare that the research was conducted in the absence of any commercial or financial relationships that could be construed as a potential conflict of interest.

## Publisher's Note

All claims expressed in this article are solely those of the authors and do not necessarily represent those of their affiliated organizations, or those of the publisher, the editors and the reviewers. Any product that may be evaluated in this article, or claim that may be made by its manufacturer, is not guaranteed or endorsed by the publisher.

## Footnotes

^1^ https://github.com/belimmer/PerturbationSaliencyEvaluation

^2^Available under: https://github.com/sicara/tf-explain.

^3^To see how these variants performed in our final insertion metric tests refer to Section 3.4.3.

^4^Let μ be the mean of the 2,000 resulting AUC values *x*_*i*_ then the SD is given by $\sqrt{\frac{\underset{i}{\sum }{\left(x_{i}-\mu \right)}^{2}}{2,000}}$.

^5^Let μ and σ be the mean and standard deviation of the mean AUC results *x*_*i*_ then the standardized results *z*_*i*_ are given by $z_{i}=\frac{x_{i}-\mu }{\sigma }$.

^6^Here the *n* percentile is the value $\frac{n}{100}$ of the way from the minimum of the similarity values to the maximum.

^7^ https://github.com/belimmer/PerturbationSaliencyEvaluation

^8^ https://github.com/belimmer/PerturbationSaliencyEvaluation

^9^For each game and metric the sample mean of the 1,000 insertion metric results *x*_*i*_ is calculated by $\mu =\frac{\underset{i}{\sum }x_{i}}{1,000}$ and the SD by $\sqrt{\frac{\underset{i}{\sum }{\left(x_{i}-\mu \right)}^{2}}{1,000}}$.
